# The development and validation of automated machine learning models for predicting lymph node metastasis in Siewert type II T1 adenocarcinoma of the esophagogastric junction

**DOI:** 10.3389/fmed.2024.1266278

**Published:** 2024-04-03

**Authors:** Chenghao Lu, Lu Liu, Minyue Yin, Jiaxi Lin, Shiqi Zhu, Jingwen Gao, Shuting Qu, Guoting Xu, Lihe Liu, Jinzhou Zhu, Chunfang Xu

**Affiliations:** ^1^Department of Gastroenterology, The First Affiliated Hospital of Soochow University, Suzhou, Jiangsu, China; ^2^Suzhou Clinical Center of Digestive Diseases, Suzhou, Jiangsu, China; ^3^Department of Gastroenterology, Beijing Friendship Hospital, Capital Medical University, National Clinical Research Center for Digestive Disease, Beijing Digestive Disease Center, State Key Laboratory of Digestive Health, Beijing, China; ^4^The Forth Affiliated Hospital of Soochow University, Suzhou, China

**Keywords:** machine learning, adenocarcinoma of the esophagogastric junction (AEG), lymph node metastasis (LNM), predictive model, Surveillance, Epidemiology, and End Results (SEER) program

## Abstract

**Background:**

Lymph node metastasis (LNM) is considered an essential prognosis factor for adenocarcinoma of the esophagogastric junction (AEG), which also affects the treatment strategies of AEG. We aimed to evaluate automated machine learning (AutoML) algorithms for predicting LNM in Siewert type II T1 AEG.

**Methods:**

A total of 878 patients with Siewert type II T1 AEG were selected from the Surveillance, Epidemiology, and End Results (SEER) database to develop the LNM predictive models. The patients from two hospitals in Suzhou were collected as the test set. We applied five machine learning algorithms to develop the LNM prediction models. The performance of predictive models was assessed using various metrics including accuracy, sensitivity, specificity, the area under the curve (AUC), and receiver operating characteristic (ROC) curve.

**Results:**

Patients with LNM exhibited a higher proportion of male individuals, a poor degree of differentiation, and submucosal infiltration, with statistical differences. The deep learning (DL) model demonstrated relatively good accuracy (0.713) and sensitivity (0.868) among the five models. Moreover, the DL model achieved the highest AUC (0.781) and sensitivity (1.000) in the test set.

**Conclusion:**

The DL model showed good predictive performance among five AutoML models, indicating the advantage of AutoML in modeling LNM prediction in patients with Siewert type II T1 AEG.

## 1 Introduction

The global incidence of adenocarcinoma of the esophagogastric junction (AEG) has been rapidly increasing (1–7). The incidence of AEG increased by 2.5 times between 1973 and 1992, according to the statistics from the National Cancer Institute’s Surveillance, Epidemiology, and End Results (SEER) program (4). A study in Japan showed that the proportion of AEG in patients with gastric adenocarcinoma increased from 2.3% (1962–1965) to 10.0% (2001–2005) (2). Similarly, an increasing trend of AEG was observed from 1988 to 2012 in a Chinese hospital (8).

AEG is commonly considered a separate digestive tract tumor (9–12). The Siewert classification categorizes AEG into three types based on the location of the tumor epicenter relative to the gastroesophageal junction (GEJ) (13–15). In Siewert type I AEG, the epicenter of the tumor is located 1 to 5 cm above the GEJ. For type II, the epicenter of the tumor is located 1 cm above to 2 cm below the GEJ. For type III, the epicenter of the tumor is located 2 to 5 cm below the GEJ. Among the three subtypes, Siewert type II is generally considered the true cardia carcinoma (13).

Due to its particular anatomical location, the treatment of Siewert type II AEG has been historically complicated. For locally advanced tumors, radical surgical resection is still the primary treatment for AEG (5, 11). However, with gastrointestinal endoscopy screening, patients with digestive tract cancer are diagnosed at an early stage, making it possible to treat early AEG without lymphatic and organ metastasis by endoscopy. The endoscopic resection of superficial AEG, such as endoscopic mucosal resection (EMR) and endoscopic submucosal dissection (ESD), is considered safe and effective (16–21). Endoscopic resection techniques are increasingly being employed for early AEG, leading to a reduction in the morbidity and mortality associated with gastrectomy or esophagectomy and an improvement in the quality of life (16).

Previous studies have shown lymph node metastasis (LNM) as an independent prognostic factor for AEG (9, 22, 23). In addition, some studies have constructed the prediction models for LNM of AEG using the traditional logistic regression method (24–27). However, machine learning-based models are increasingly used in the diagnosis, prediction, and prognosis evaluation of gastrointestinal diseases, such as inflammatory bowel disease and gastrointestinal tumors (28–31). In this study, we aimed to establish predictive models for LNM in Siewert type II T1 AEG using automated machine learning (AutoML) methods to help clinicians assess the availability of endoscopic treatment and individualize a suitable treatment for patients.

## 2 Materials and methods

### 2.1 Data source

Relevant data from the SEER database were retrieved in our study. The SEER database of the National Cancer Institute, an authoritative source of information on cancer incidence and survival, contains data on various tumor sites and from sources throughout the United States.^1^ Currently, the SEER program collects and releases cancer data from 17 population-based registries, covering approximately one-third of the U.S. population, which can be used to conduct population-based case–control studies that clarify the etiology of cancers, especially some uncommon ones (32, 33). By using SEER ∗ Stat 8.4.0.1 software, we obtained demographic information and cancer incidence data collected from the SEER 17 Registries, November 2021 Sub (2000–2019 varying). To identify Siewert type II AEG, we used two parameters in the SEER database. Cancers simultaneously satisfying two conditions [“TNM 7/CS v0204 + Schema” encoded 28 (Esophagus GE Junction) and “Primary Site-Labeled” encoded 160 (Cardia, NOS)] were extracted and classified as Siewert type II AEG (4, 34).

In addition, the patients with Siewert type II T1 AEG diagnosed in the First Affiliated Hospital of Soochow University and the Second Affiliated Hospital of Soochow University from April 2003 to October 2022 were retrospectively selected as the research subjects.

### 2.2 Including criteria

The criteria for patient inclusion were as follows: (1) patients with available TNM stage information; (2) patients aged 18 years or above at diagnosis (in consideration of the tiny proportion of patients under 18 years); (3) patients pathologically diagnosed as T1M0 Siewert Type II AEG; (4) patients with the first or only primary malignancy; and (5) patients with available information on differentiation, extension, and size.

### 2.3 The development of models

#### 2.3.1 Variable selection and data pre-processing

Patient demographics (age, sex, race, year of diagnosis, and marital status) and tumor characteristics (tumor grade, tumor size, T stage, N stage, M stage, number of lymph nodes examined, and number of positive lymph nodes) were collected from the SEER database and the hospitals in Suzhou. We finally obtained eight variables (age, race, sex, marital status, differentiation, extension, size, and LNM) for our analysis.

Missing values for variables can complicate data analysis and introduce potential bias into the final results. In this study, classification variables that could not be evaluated after missing value analysis were removed. Missing interpolation was performed on continuous variables, such as tumor size, using the multivariate imputation by chained equations package (MICE version 3.15.0) in R, version 4.2.2 (Institute for Statistics and Mathematics, Vienna, Austria; http://www.r-project.org), based on the random forest method (35). The SEER data were randomly divided into the training (*n* = 629) and validation (*n* = 238) sets in a ratio of 7:3. A total of 141 samples from Suzhou were used as the test set.

The distribution of LNM outcomes was imbalanced in the training and validation sets, leading to particular bias in modeling and evaluation of model performance. To address this issue, we used the SMOTE function in the DMwR package (version 0.4.1) in R to balance the training set and the validation set by applying undersampling and oversampling techniques. Finally, we got the balanced training set (*n* = 949) and validation set (*n* = 380).

#### 2.3.2 Modeling methods and evaluation

In this study, five algorithms—generalized linear model (GLM), gradient boosting machine (GBM), deep learning (DL), distributed random forest (DRF), and stacked ensemble (SE)—were provided by H2O^2^ to construct prediction models using the training set. Using the h2o package (version 3.38.0.4) in R, we set the response column and the predictor columns for the training, validation, and test sets, respectively. The H2O AutoML performs a hyperparameter search using a random grid search method over the five algorithms to deliver the best model automatically. Five predictive models were finally developed for this approach. We used the validation and test sets to score and rank models.

**Figure 1:**
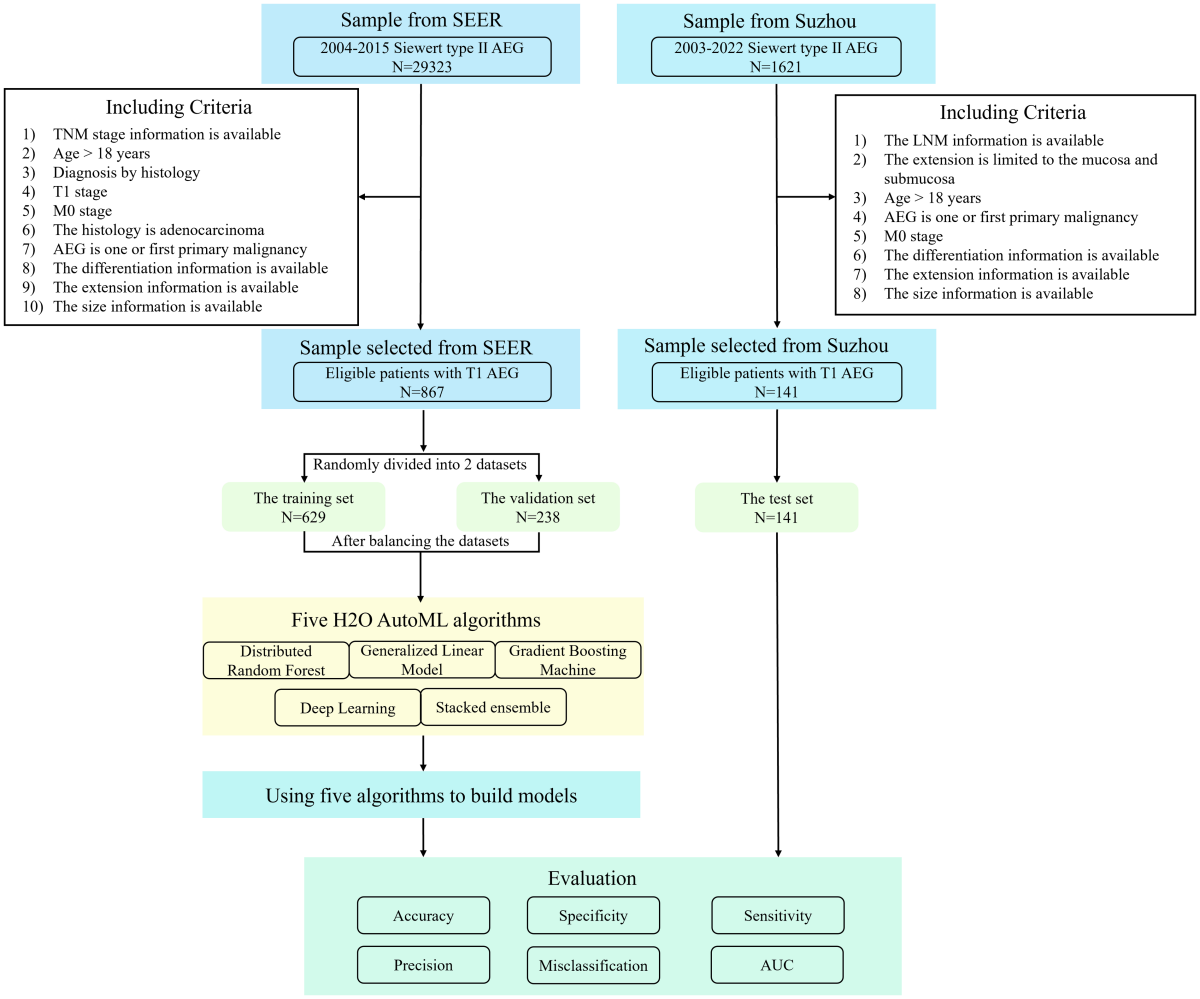
Flowchart of patient selection and modeling procedure.

The models’ accuracy, misclassification, specificity, sensitivity, and precision (also named positive predictive value) were obtained by plotting the confusion matrix. To select the best model, the difference between the predicted and actual results was analyzed. The predictive ability of the models was evaluated using the receiver operating characteristics (ROC) curve and the area under the curve (AUC). The procedure of patient selection and modeling is shown in Figure 1.

We selected a model with the best performance using the above indicators and further evaluated it with the calibration curve. The Brier score, a statistical metric to measure the accuracy of probabilistic forecasts, was used to assess the calibration degree of the models. The score ranges from 0 to 1; a model with perfect skill has a score of 0, and the poorest model has a score of 1 (36). The unreliability index and the *p*-value of the calibration curve were also used to evaluate the reliability of the model.

Finally, the results of the model are presented visually for better understanding. A variable importance plot was constructed to show the importance of different variables. A Local Interpretable Model-Agnostic Explanations (LIME) Feature Importance Visualization plot was constructed using the lime package (version 0.5.3) in R to show the contributions of variables of samples to the outcome.

### 2.4 Statistical analysis

The statistical analysis and the modeling process were performed using R software. The package, tableone (version 0.13.2), in R was used in data analysis. We compared the baseline information and characteristics between different groups, including demographic and clinicopathological data. The normality of the quantitative data was evaluated using the Kolmogorov–Smirnov test. When the quantitative variables were normally distributed, they were represented by the mean and standard deviation (SD). However, they were represented by the median and interquartile range (IQR) when they were not. Student’s t-test was used for intergroup comparison of normally distributed quantitative variables, and the Mann–Whitney U-test was employed to compare non-normally distributed quantitative variables. The classification data were expressed by frequency and percentage, and the chi-squared (χ2) test was used for intergroup comparison.

## 3 Results

### 3.1 Characteristics

A total of 867 patients from the SEER dataset and 141 patients from Suzhou were screened for our study. After balancing the SEER dataset, 1,329 samples were collected in the SEER dataset. The patients’ demographics and clinicopathological baseline information in the SEER dataset after balancing and the test set are summarized, respectively, in Table 1. The baseline characteristic information based on LNM in the SEER dataset after balancing and the test set is summarized in Tables 2, 3, respectively. In the SEER dataset, the median sizes of the LNM and non-LNM groups were 25.00 and 15.00 mm, respectively, with statistical significance (*p* < 0.001). Patients with LNM had a higher proportion of poor degree of differentiation and submucosal infiltration, with statistical differences. In the test set, the average ages of the LNM and non-LNM groups were 65.00 and 67.15 years, respectively, with no statistical significance (*p* = 0.344). Patients with LNM had a higher proportion of poor degree of differentiation, with a p-value of 0.051. Patients in the two groups showed statistical differences in the depth of tumor invasion.

**Table 1:** Baseline characteristics of patients from the SEER dataset after balancing and the test set.

	SEER dataset	The test set	*P*-value
Age (year)			0.493
Median	66.00	67.00	
Interquartile range	14.00	8.00	
Race			<0.001
American Indian	5 (0.4%)	0 (0.0%)	
Asian or Pacific Islander	97 (7.3%)	141 (100.0%)	
Black	84 (6.3%)	0 (0.0%)	
White	1,134 (85.3%)	0 (0.0%)	
Unknown	9 (0.7%)	0 (0.0%)	
Sex			0.062
Female	340 (25.6%)	26 (18.4%)	
Male	989 (74.4%)	115 (81.6%)	
Marriage			<0.001
Divorced	135 (10.2%)	0 (0.0%)	
Married	789 (59.4%)	140 (99.3%)	
Separated	8 (0.6%)	0 (0.0%)	
Never married	143 (10.8%)	0 (0.0%)	
Widow	205 (15.4%)	1 (0.7%)	
Unknown	49 (3.7%)	0 (0.0%)	
Differentiation^1^			0.012
1 (well)	163 (12.3%)	18 (12.8%)	
2 (moderately)	663 (49.9%)	87 (61.7%)	
3 (poorly)	503 (37.8%)	36 (25.5%)	
Extension^2^			0.721
Intramucosal	539 (40.6%)	55 (39.0%)	
Submucosal	790 (59.4%)	86 (61.0%)	
Tumor size (mm)			0.584
Median	20.00	20.00	
Interquartile range	19.00	17.50	
LNM^3^			<0.001
0	667 (50.2%)	131 (92.9%)	
1	662 (49.8%)	10 (7.1%)	

^1^For the differentiation variable, well-differentiated is defined as 1, moderately differentiated is defined as 2, and poorly differentiated or undifferentiated is defined as 3. ^2^The variable extension refers to the depth of tumor invasion. ^3^For the LNM variable, those with lymph node metastases are defined as 1; the rest are defined as 0.

**Table 2:** Clinicopathological characteristics of patients from the SEER dataset after balancing.

	Total	Non-LNM	LNM	*P*-value
Age (year)				0.216
Median	66.00	67.00	66.00	
Interquartile range	14.00	17.00	13.00	
Race				<0.001
American Indian	5 (0.4%)	1 (0.1%)	4 (0.6%)	
Asian or Pacific Islander	97 (7.3%)	36 (5.4%)	61 (9.2%)	
Black	84 (6.3%)	25 (3.7%)	59 (8.9%)	
White	1,134 (85.3%)	601 (90.1%)	533 (80.5%)	
Unknown	9 (0.7%)	4 (0.6%)	5 (0.8%)	
Sex				0.337
Female	340 (25.6%)	163 (24.4%)	177 (26.7%)	
Male	989 (74.4%)	504 (75.6%)	485 (73.3%)	
Marriage				<0.001
Divorced	135 (10.2%)	59 (8.8%)	76 (11.5%)	
Married	789 (59.4%)	436 (65.4%)	353 (53.3%)	
Separated	8 (0.6%)	6 (0.9%)	2 (0.3%)	
Never married	143 (10.8%)	67 (10.0%)	76 (11.5%)	
Widow	205 (15.4%)	69 (10.3%)	136 (20.5%)	
Unknown	49 (3.7%)	30 (4.5%)	19 (2.9%)	
Differentiation^1^				<0.001
1 (well)	163 (12.3%)	116 (17.4%)	47 (7.1%)	
2 (moderately)	663 (49.9%)	364 (54.6%)	299 (45.2%)	
3 (poorly)	503 (37.8%)	187 (28.0%)	316 (47.7%)	
Extension^2^				0.001
Intramucosal	539 (40.6%)	300 (45.0%)	239 (36.1%)	
Submucosal	790 (59.4%)	367 (55.0%)	423 (63.9%)	
Tumor size (mm)				<0.001
Median	20.00	15.00	25.00	
Interquartile range	19.00	17.00	18.00	

^1^For the differentiation variable, well-differentiated is defined as 1, moderately differentiated is defined as 2, and poorly differentiated or undifferentiated is defined as 3. ^2^The variable extension refers to the depth of tumor invasion.

**Table 3:** Clinicopathological characteristics of patients from the test set.

	Total	Non-LNM	LNM	*P*-value
Age (year)				0.344
Average	66.48	67.15	65.00	
Standard deviation	10.87	6.98	5.75	
Sex				1.000
Female	26 (18.4%)	24 (18.3%)	2 (20.0%)	
Male	115 (81.6%)	107 (81.7%)	8 (80.0%)	
Marriage				1.000
Divorced	0 (0.0%)	0 (0.0%)	0 (0.0%)	
Married	140 (99.3%)	130 (99.2%)	10 (100.0%)	
Separated	0 (0.0%)	0 (0.0%)	0 (0.0%)	
Never married	0 (0.0%)	0 (0.0%)	0 (0.0%)	
Widow	1 (0.7%)	1 (0.8%)	0 (0.0%)	
Unknown	0 (0.0%)	0 (0.0%)	0 (0.0%)	
Differentiation^1^				0.051
1 (well)	18 (12.8%)	18 (13.7%)	0 (0.0%)	
2 (moderately)	87 (61.7%)	83 (63.4%)	4 (40.0%)	
3 (poorly)	36 (25.5%)	30 (22.9%)	6 (60.0%)	
Extension^2^				0.007
Intramucosal	55 (39.0%)	55 (42.0%)	0 (0.0%)	
Submucosal	86 (61.0%)	76 (58.0%)	10 (100.0%)	
Tumor size (mm)				0.184
Median	20.00	20.00	22.50	
Interquartile range	17.50	18.00	32.00	

^1^For the differentiation, well-differentiated is defined as 1, moderately differentiated is defined as 2, and poorly differentiated or undifferentiated is defined as 3. ^2^The variable extension refers to the depth of tumor invasion.

The clinicopathological features of the training set and the validation set obtained from the SEER dataset are presented in Table 4. In the balanced training set and validation set, the rate of positive events, just the LNM rate in the study, was 49.7% and 50%, respectively. There were no significant differences in gender, degree of differentiation, depth of tumor invasion, or tumor size between the two groups.

**Table 4:** Clinicopathological characteristics of patients from the training set and the validation set after balancing.

	The training set	The validation set	*P*-value
Age (year)			0.014
Median	66.00	66.00	
Interquartile range	13.00	17.00	
Race			<0.001
American Indian	5 (0.5%)	0 (0.0%)	
Asian or Pacific Islander	45 (4.7%)	52 (13.7%)	
Black	63 (6.6%)	21 (5.5%)	
White	830 (87.5%)	304 (80.0%)	
Unknown	6 (0.6%)	3 (0.8%)	
Sex			0.279
Female	235 (24.8%)	105 (27.6%)	
Male	714 (75.2%)	275 (72.4%)	
Marriage			0.007
Divorced	80 (8.4%)	55 (8.4%)	
Married	582 (61.3%)	207 (54.5%)	
Separated	8 (0.8%)	0 (0.0%)	
Never married	98 (10.3%)	45 (11.8%)	
Widow	147 (15.5%)	58 (15.3%)	
Unknown	34 (3.6%)	15 (3.9%)	
Differentiation^1^			0.569
1 (well)	122 (12.9%)	41 (10.8%)	
2 (moderately)	472 (49.7%)	191 (50.3%)	
3 (poorly)	355 (37.4)	148 (38.9%)	
Extension^2^			0.467
Intramucosal	379 (39.9%)	160 (42.1%)	
Submucosal	570 (60.1%)	220 (57.9%)	
Tumor size (mm)			0.491
Median	20.00	20.00	
Interquartile range	19.00	19.00	
LNM^3^			0.931
0	477 (50.3%)	190 (50.0%)	
1	472 (49.7%)	190 (50.0%)	

^1^For the differentiation variable, well-differentiated is defined as 1, moderately differentiated is defined as 2, and poorly differentiated or undifferentiated is defined as 3. ^2^The variable extension refers to the depth of tumor invasion. ^3^For the LNM variable, those with lymph node metastases are defined as 1; the rest are defined as 0.

### 3.2 Performance of the models

**Figure 2:**
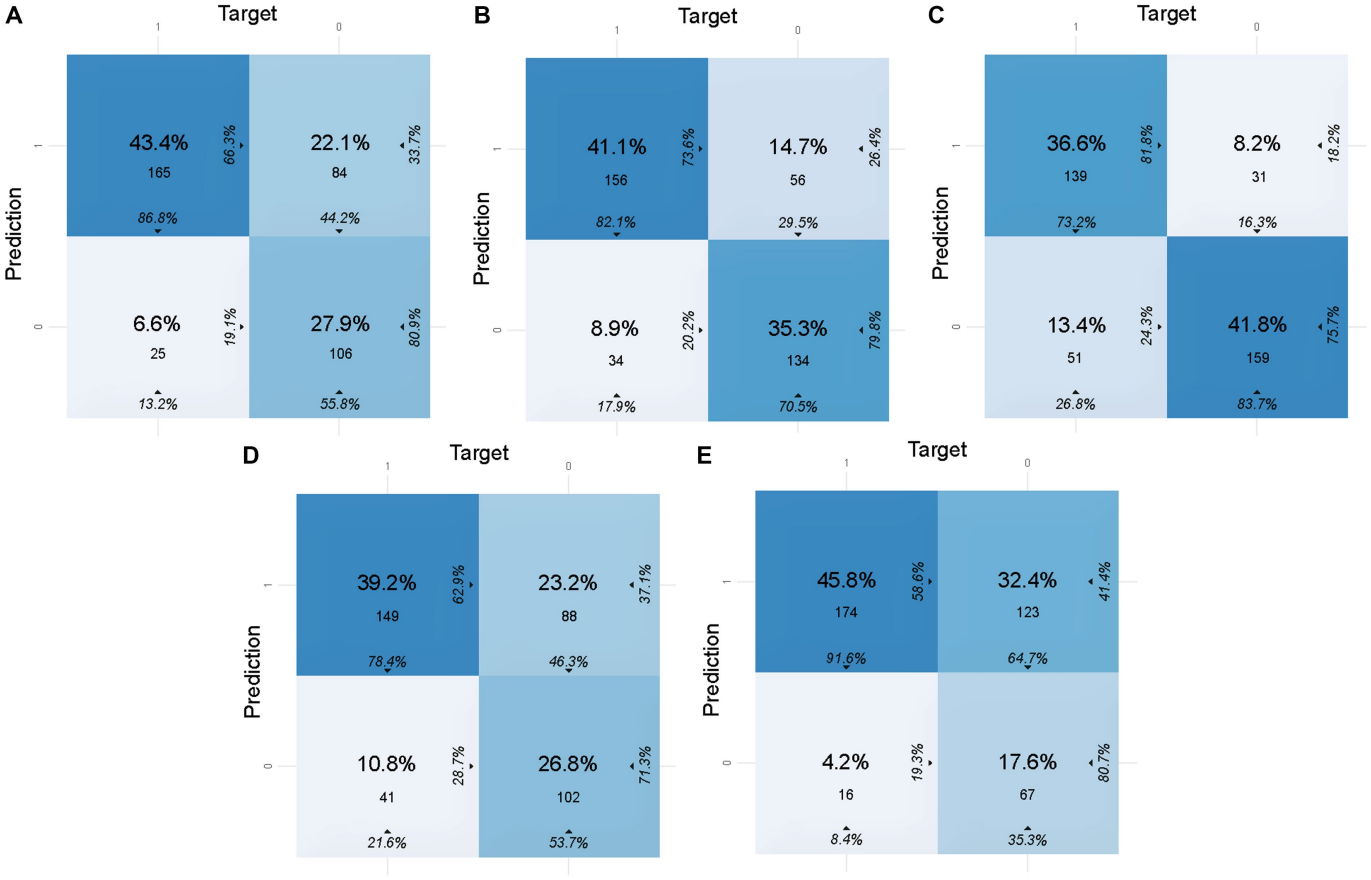
Confusion matrices of five models in the validation set. In this figure, 1 of target represents lymph node metastasis in the population, while 1 of prediction represents the positive prediction of lymph node metastasis by the model. **(A)** Confusion matrix of the DL model in the validation set. **(B)** Confusion matrix of the GBM model in the validation set. **(C)** Confusion matrix of the SE model in the validation set. **(D)** Confusion matrix of the DRF model in the validation set. **(E)** Confusion matrix of the GLM model in the validation set.

**Figure 3:**
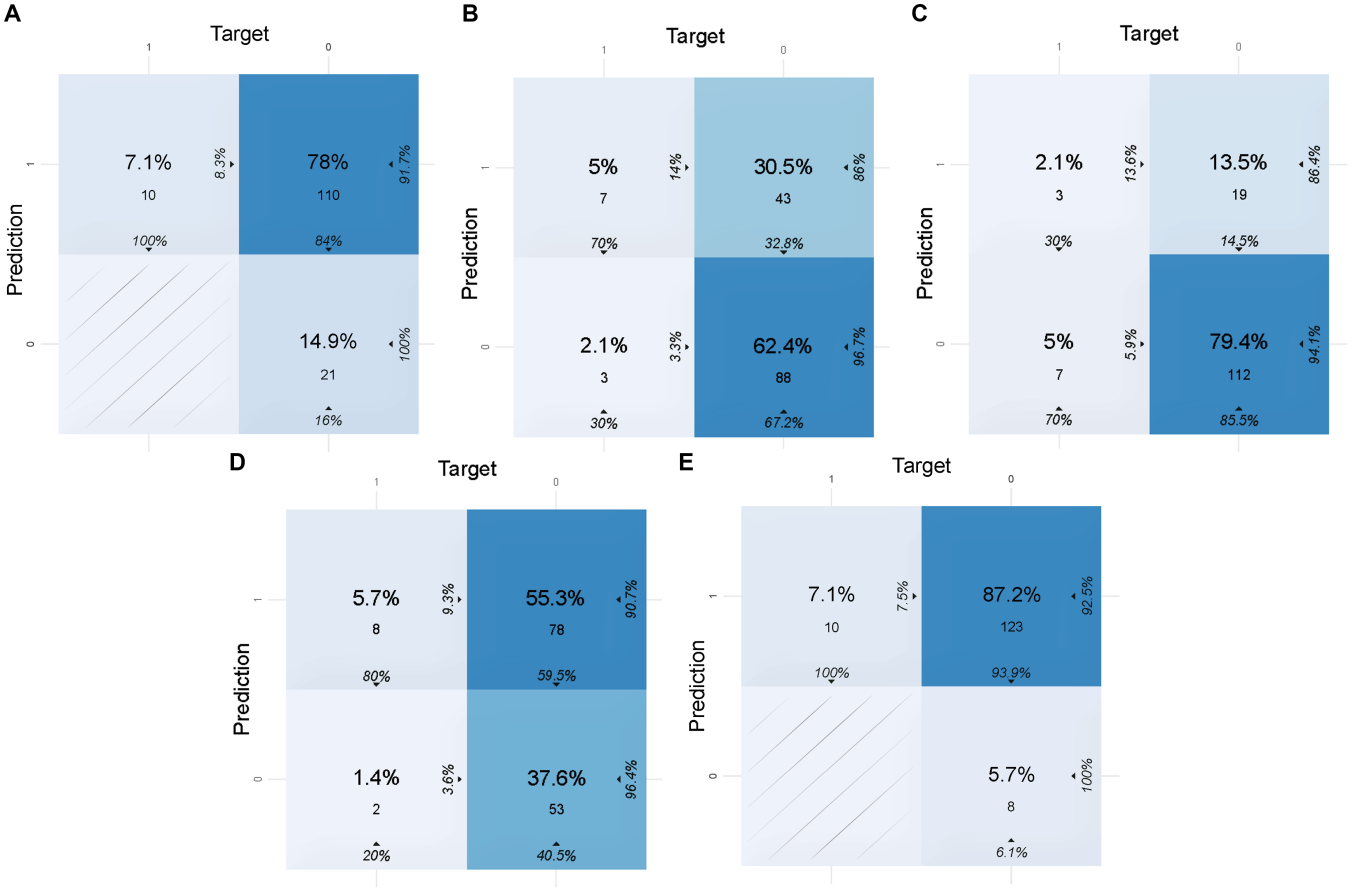
Confusion matrices of five models in the test set. In this figure, 1 of target represents lymph node metastasis in the population, while 1 of prediction represents the positive prediction of lymph node metastasis by the model. **(A)** Confusion matrix of the DL model in the test set. **(B)** Confusion matrix of the GBM model in the test set. **(C)** Confusion matrix of the SE model in the test set. **(D)** Confusion matrix of the DRF model in the test set. **(E)** Confusion matrix of the GLM model in the test set.

To calculate the accuracy, sensitivity, specificity, and other indicators of the models in the validation set and the test set, the confusion matrices of the models are shown in Figures 2, 3, respectively. These indicators and the AUC of the five different models in the validation and test sets are shown in Table 5. The DL model has good sensitivity (0.868) and accuracy (0.713) in the validation set, which means that the model can accurately identify patients with positive lymph node metastases. The DL model exhibited a sensitivity of 100% in the test set, indicating that the model was able to screen out node-positive patients well and reduce missed diagnoses. The GBM model achieved an accuracy of 0.763, a sensitivity of 0.821, and a specificity of 0.705 in the validation set. The sensitivity (0.700) of the model in the test is lower than that of the DL model. The confusion matrix revealed that the model failed to correctly predict three patients with positive lymph nodes in the test set. Although the GLM model exhibited the highest sensitivity in the validation set (0.916) and the test set (1.000), its specificity was lower than other models. This aspect suggests that the GLM model was less capable of predicting negative LNM.

**Table 5:** Performance of models in the dataset.

	Accuracy	Misclassification	Sensitivity	Specificity	Precision	AUC
**The validation set**						
DL	0.713	0.287	0.868	0.558	0.663	0.769
GBM	0.763	0.237	0.821	0.705	0.736	0.840
SE	0.784	0.216	0.732	0.837	0.818	0.836
DRF	0.661	0.339	0.784	0.537	0.629	0.718
GLM	0.634	0.366	0.916	0.353	0.586	0.752
**The test set**						
DL	0.220	0.780	1.000	0.160	0.083	0.781
GBM	0.674	0.326	0.700	0.672	0.140	0.726
SE	0.816	0.184	0.300	0.855	0.136	0.633
DRF	0.433	0.567	0.800	0.405	0.093	0.582
GLM	0.128	0.872	1.000	0.061	0.075	0.766

DL, deep learning; GBM, gradient boosting machine; SE, stacked ensemble; DRF, distributed random forest; GLM, generalized linear model.

**Figure 4:**
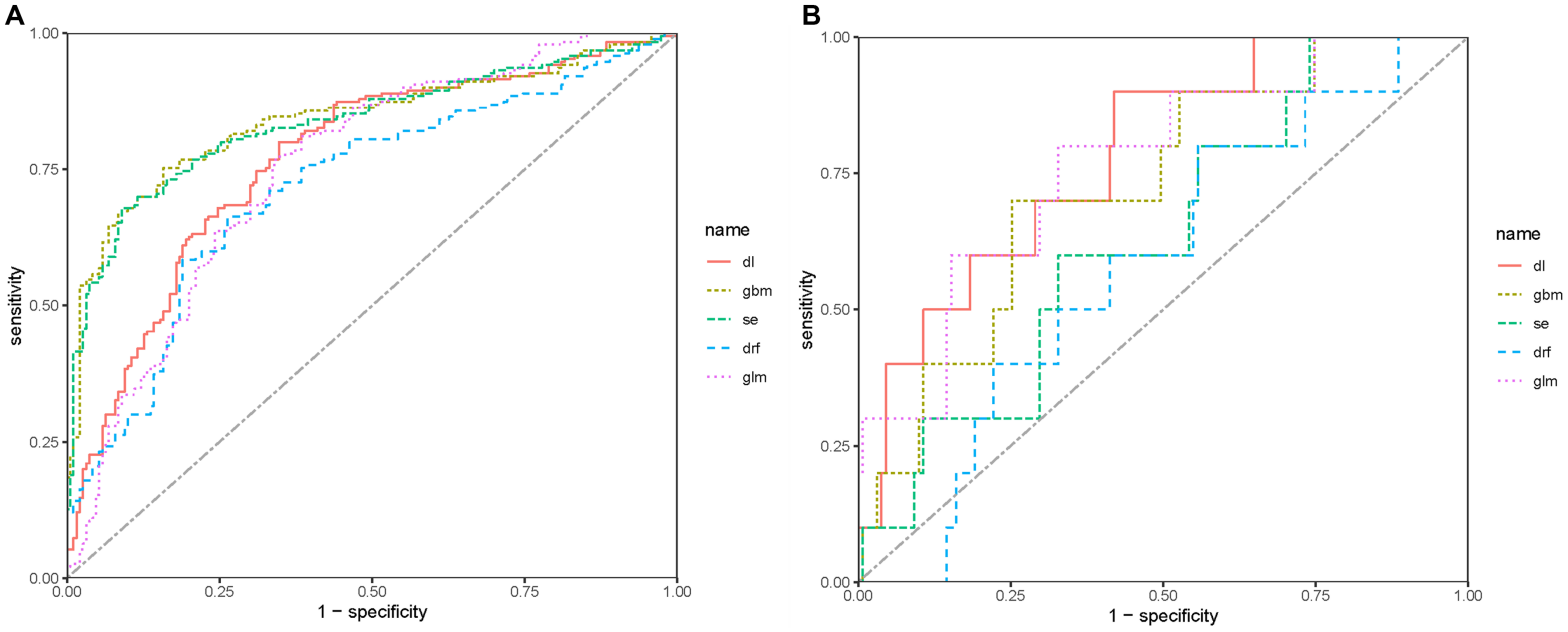
ROC of predictive models in the sets. **(A)** ROC of predictive models in the validation set. **(B)** ROC of predictive models in the test set.

The ROC curve and AUC can evaluate the predictive ability of the models. Figures 4A,B show the ROC curves of the five models in the validation and test sets, respectively. The DL model achieved a good AUC (0.769) in the validation set, and it exhibited the highest AUC in the test set compared to other models. The Matthews correlation coefficient (MCC) score is a commonly used metric for evaluating binary classification models. The DL model achieved a MCC score of 0.448. The MCC scores of the five predictive models on the test set are not high, which may be related to the small size of the test set.

Considering that the predictive model is a preoperative screening model, sensitivity should have a high weight on the selection of models. Hence, we believe that the DL model is the best model for predicting LNM in patients with AEG, with high sensitivity and reasonable specificity. The DL model consists of an input layer, two hidden layers, and an output layer. Dropout is applied in both hidden layers at a rate of 30%, which helps to prevent overfitting by randomly dropping out a percentage of units during modeling. The regularization terms are set to 0 for all layers. Rectifier (ReLU) activation functions are used in the hidden layers, while Softmax activation is used in the output layer for classification.

### 3.3 The performance of the deep learning model

#### 3.3.1 Calibration curve in the datasets

**Figure 5:**
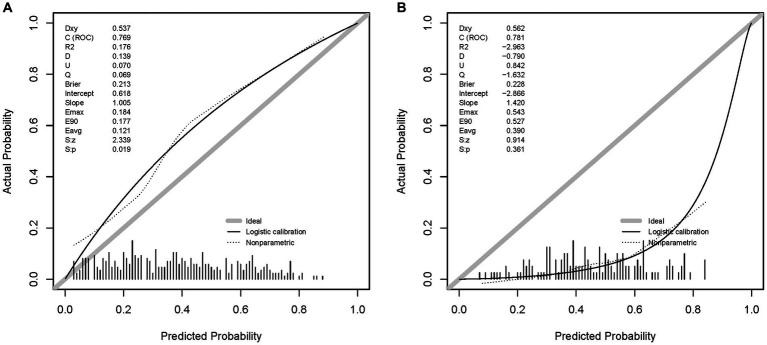
Calibration curves of the DL model in the sets. **(A)** The calibration curve of the DL model in the validation set. **(B)** The calibration curve of the DL model in the test set.

The calibration curves are shown in Figures 5A,B, which is another way to evaluate the model. The calibration curve of the DL model in the validation set shows a high degree of fit. The Brier scores of the DL model in the validation and test sets were 0.213 and 0.228, respectively, indicating that the prediction results of the model were in good agreement with the actual outcome. The unreliability index of the model in the validation set was 0.070, which suggests that the DL model is reliable for predicting the LNM in T1 Siewert type II AEG patients.

#### 3.3.2 Model visualization

**Figure 6:**
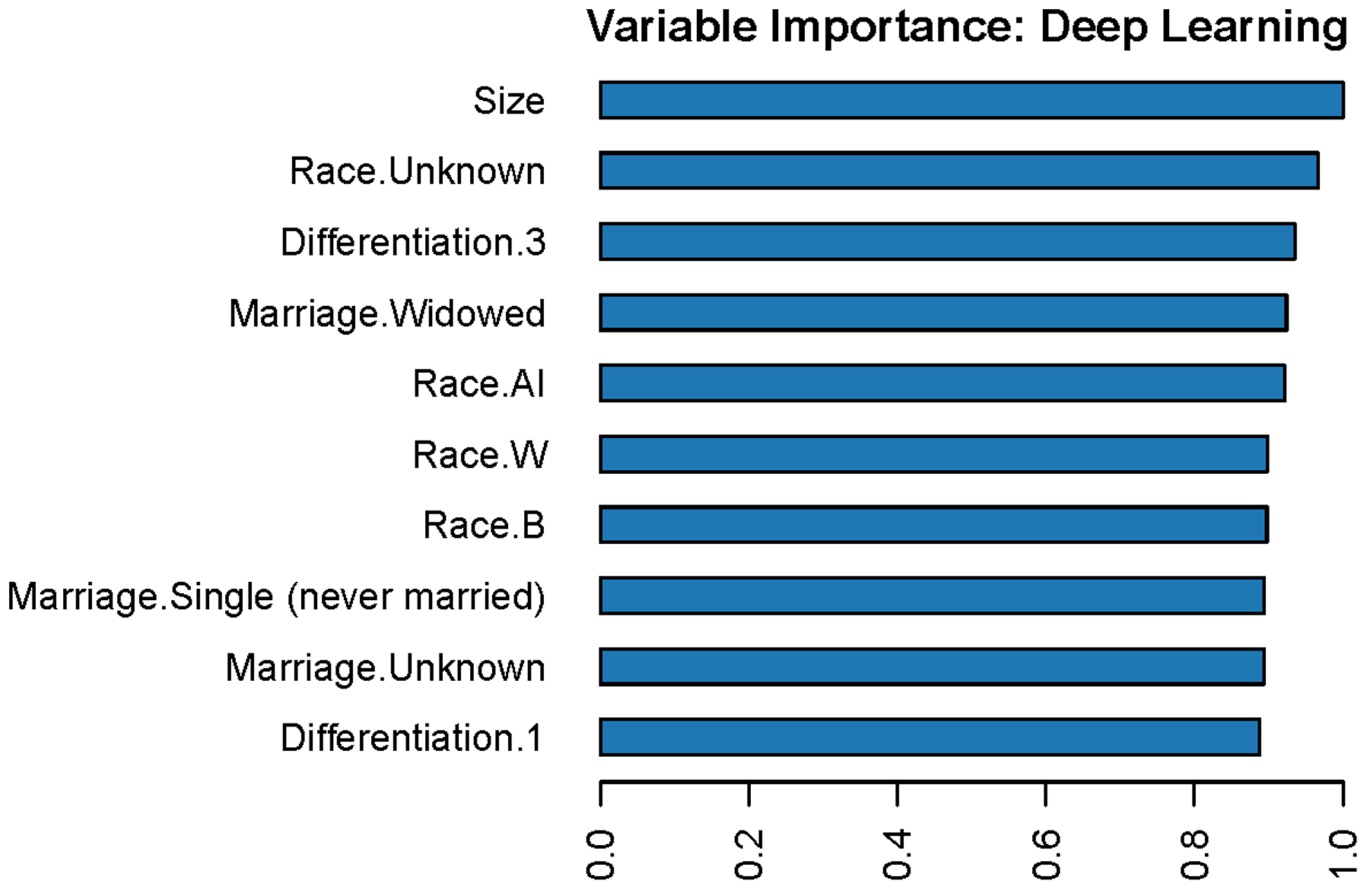
Variable importance in the DL model. In this figure, “Race.AI” means patients’ race is American Indian; “Race.W” means patients’ race is white; “Race.B” means patients’ race is Black; “Differentiation.1” means well-differentiated tumors; “Differentiation.3” means poorly differentiated tumors.

**Figure 7:**
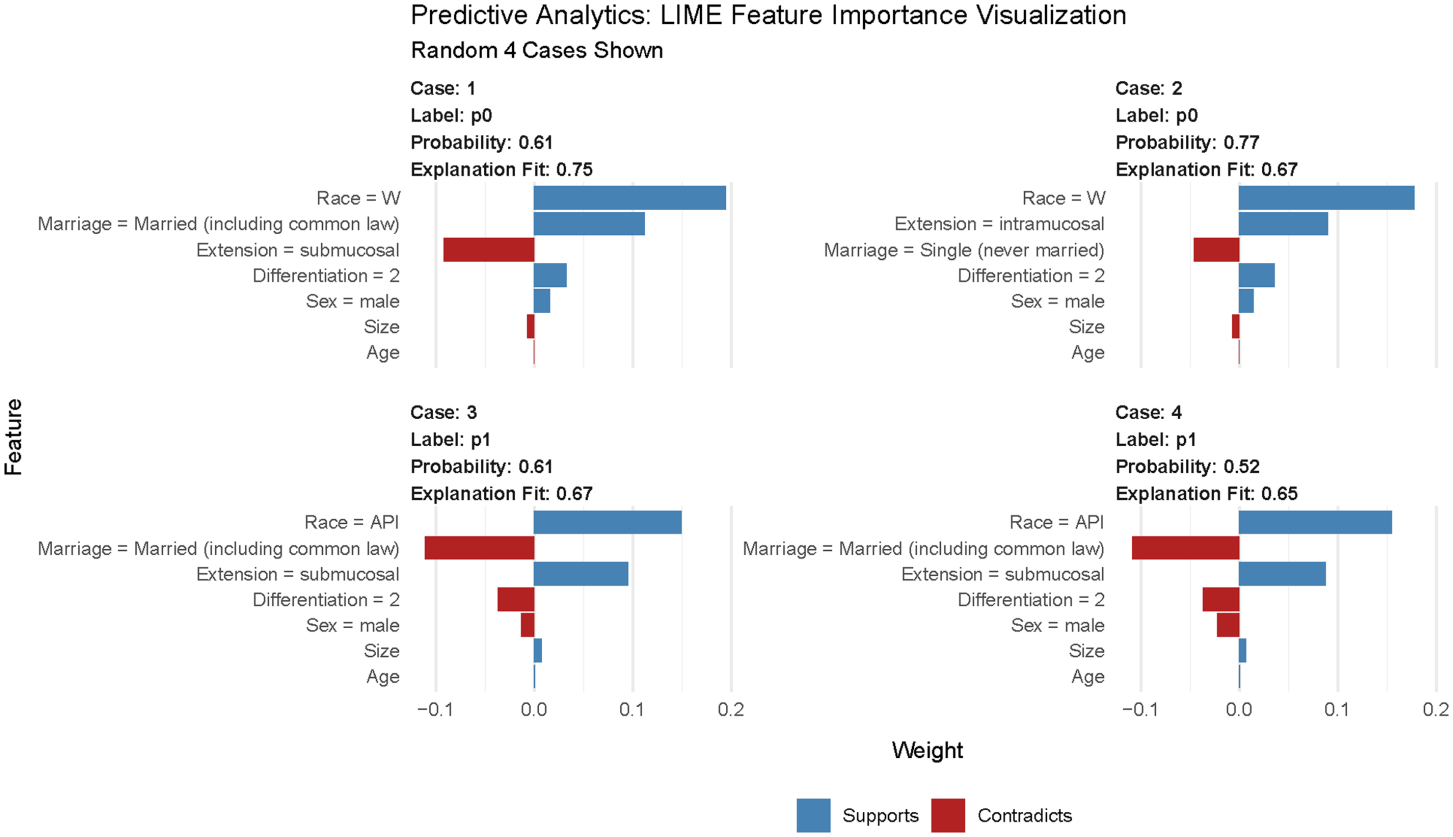
LIME feature importance visualization. In this figure, “Race = W” means patients’ race is white; “Race = API” means patients’ race is Asian or Pacific Islander; “Differentiation = 2” means tumor is moderately differentiated.

The variable importance in the DL model is shown in Figure 6. According to this figure, tumor size is the most important predictor of LNM in T1 Siewert type II patients. Furthermore, we randomly selected four cases to plot the LIME feature importance visualization, as shown in Figure 7. Take the first case as an example, the tumor is moderately differentiated, and the white male married patient supports the lymph node without metastasis. In the third case, male married patients with moderately differentiated tumors contradict the result of LNM. However, other parameters, such as tumor infiltration into the submucosa and patients from Asian or Pacific regions, support LNM. Under the comprehensive prediction, the probability of LNM in this patient was 61%. That is, the prediction result is the same as the actual outcome.

## 4 Discussion

In the study, we found that differentiation, the depth of invasion, the size of the AEG, and gender were related to LNM. Five predictive models were developed using AutoML. Among these models, the DL model is the most suitable for predicting and screening LNM in early AEG, with the highest sensitivity and AUC in the test set.

With the incidence rates rising, a series of problems in the treatment and prognosis of AEG have been gradually becoming global concerns. With the application of endoscopic screening technology, patients with digestive tract cancer (including AEG) are diagnosed at an early stage, making endoscopic treatment of superficial AEG possible. Because of the inherent differences in the anatomy of AEG, there are certain technical difficulties in treating AEG with ESD (37). Chen et al. (38) found that the procedure speed of ESD for early AEG is slower than that for early gastric carcinoma, possibly due to AEG extending beyond the cardia, including the angle of His. However, endoscopic treatment (including ESD) remains an effective alternative to surgery for the treatment of early AEG based on comparable long-term outcomes (18, 20). With the advancements in endoscopic treatment, early AEG can be effectively resected by EMR/ESD with fewer complications, better preservation of gastric function, a shorter duration of hospital stay, and a lower cost compared with traditional gastrectomy or esophagectomy (16, 17). Endoscopic treatment is gaining acceptance because it is more tolerable, especially in elderly patients. Chen et al. (20) revealed that endoscopic treatment may be considered in patients aged 65 years or those with submucosal (T1b stage) cancer of the AEG.

It has been widely accepted that LNM is an important prognostic factor for patients with Siewert type II AEG. In the study of Wang et al. (22), a prognostic model for the outcome of patients with AEG based on a traditional algorithm was established. The positive lymph nodes and the ratio of metastatic lymph nodes were identified as two of the prognostic factors according to the univariate analysis. Naoki et al. (23) found that LNM was the only independent prognostic factor for AEG in their study. To achieve better endoscopic treatment effects, early AEG should meet certain standards (including no lymph node and distant organ metastasis) (12, 16, 18). In some studies, the location of the LNM has been found to have a significant impact on the surgical method and the scope of lymph node dissection (39–41).

Preoperative diagnosis of LNM mainly relies on computed tomography (CT), endoscopic ultrasound, and magnetic resonance imaging (MRI), which are primarily based on the size of the lymph nodes. The preoperative prediction of LNM using the CT criteria has high specificity (23). However, the diagnostic accuracy of LNM prediction using these methods is not particularly high, as the evaluation of the lymph node size is greatly affected by other factors and thus heavily relies on the physician’s evaluation (26). In addition, detecting LNM in a narrow space (such as the diaphragm, aorta, and pericardium) by contrast-enhanced CT before surgery is more complex than in lymph nodes around the stomach or colon (23). Moreover, not all patients have access to contrast-enhanced CT for diagnosing clinical LNM.

Several studies have constructed LNM predictive models of AEG until now. However, most studies only used traditional logistic regression analysis for risk factors and did not perform independent external validation. Chen et al. (25) used the logistic regression method to predict the LNM risk in early AEG patients, and the AUC of the prediction model is 0.742. Feng et al. (26) provided a detailed explanation of the correlation between tumor size and LNM in AEG and used logistic regression to plot a nomogram, which can predict the LNM risk. Zheng et al. (24) used small samples to explore the risk factors for LNM in AEG while showing the specific groups of lymph nodes. All of these studies are consistent with our findings, but the predictive performance of their models is weaker than that of ours, indicating that machine learning has good advantages in the establishment of LNM predictive models.

In the present study, a SEER-based case–control analysis has been conducted. We found that most AEG patients (approximately 71.2%) with LNM had a submucosal invasion. Approximately 53.9% of patients without LNM had submucosal infiltration. Given the key role of LNM in the selection of endoscopic or surgical resection, we built predictive models of LNM using AutoML methods on data from the SEER database and validated the models with independent data. Among the five models, the DL model is highly sensitive to predict LNM in early AEG patients. A consistent performance of our new DL model across the datasets with different baseline characteristics provides evidence of its robustness and generalizability.

In our study, we found that the degree of tumor differentiation, the depth of tumor invasion, and gender were related to LNM. Lower degrees of differentiation have higher incidences of LNM. Higher incidences of LNM are observed in less differentiated tumors due to higher heterogeneity and more aggressive biological characteristics compared to other histological types. The risk of LNM is higher for AEG that invades the submucosa. The reason behind this observation may be due to the presence of substantial lymphatic capillaries in the submucosa and the large gap between adjacent endothelial cells. If the tumor infiltrates the submucosa or deeper, cancer cells could invade the lymphatic capillaries, resulting in LNM (24). In terms of gender, the exact reason remains unknown. However, several studies have shown more prolonged survival in female individuals than in male counterparts with esophageal cancer (42–45), which is attributed to both sex itself (sex hormones and reproductive factors) and other extrinsic risk factors (43).

The predictive model we have established can help clinicians predict the LNM risk of early AEG while combining imaging findings, thus helping us make better clinical decisions and personalized treatment plans for early AEG patients. Of note, the prediction model was developed using postoperative pathological data, which can also be obtained from endoscopically resected pathological specimens. Hence, we investigated LNM predictive models for T1 AEG. However, certain limitations still exist in our study. First, the inherent limitations of retrospective and non-randomized studies may lead to unavoidable bias. Second, the prediction model was based on postoperative pathological data, and therefore, further studies combined with preoperative data are needed to validate our model. Third, the patient data from AEG were collected from two hospitals in Suzhou to validate our prediction models. Due to the low rate of LNM in the population, the test set is highly imbalanced, with positive cases representing less than 10% of cases, which makes it less credible to validate the models’ predictive performance in the test set. That is why the precision of the model in the test set is not ideal, which means data from different hospitals in different regions need to be further collected to expand the sample size. Lymphovascular invasion has been repeatedly demonstrated as the most crucial risk factor for LNM (46). The esophageal invasion length is thought to be associated with mediastinal LNM (39, 47, 48). However, due to the limited data available in the SEER database, certain tumor characteristics (such as lymphovascular invasion, esophageal invasion length, and the groups of LNM), blood index, and imaging data were missing. Therefore, we cannot further improve the performance of the LNM predictive model in a multimodal way.

## 5 Conclusion

In summary, in this multicenter-based case–control study, we report that the degree of tumor differentiation, tumor size, gender, and depth of tumor invasion are correlated with the LNM of Siewert type II T1 AEG. Using AutoML algorithms, we built five models to predict LNM in the early AEG. The DL model is the best model for predicting LNM in patients with AEG, with high sensitivity and reasonable specificity. This model should be further applied in clinical practice, and the predictive performance of this model should be prospectively explored in further clinical follow-up.

## Data availability statement

The raw data supporting the conclusions of this article will be made available by the authors, without undue reservation.

## Ethics statement

The studies involving humans were approved by the Ethics Committee of the First Affiliated Hospital of Soochow University. The studies were conducted in accordance with the local legislation and institutional requirements. Written informed consent for participation was not required from the participants or the participants’ legal guardians/next of kin in accordance with the national legislation and institutional requirements.

## Author contributions

CL: Formal analysis, Investigation, Methodology, Resources, Software, Validation, Writing – original draft. LuL: Validation, Writing – review & editing. MY: Validation, Visualization, Writing – review & editing. JL: Data curation, Methodology, Writing – review & editing. SZ: Methodology, Software, Writing – review & editing. JG: Data curation, Methodology, Writing – review & editing. SQ: Data curation, Validation, Writing – review & editing. GX: Validation, Writing – review & editing. LiL: Data curation, Validation, Writing – review & editing. JZ: Conceptualization, Funding acquisition, Supervision, Writing – review & editing. CX: Conceptualization, Funding acquisition, Project administration, Writing – review & editing.

## Footnotes

^1^ https://seer.cancer.gov/

^2^ https://h2o.ai/
