# Supplementary material for: The development and validation of automated machine learning models for predicting lymph node metastasis in Siewert type II T1 adenocarcinoma of the esophagogastric junction
Source: Front Med (Lausanne). 2024 Apr 3;11:1266278. doi: 10.3389/fmed.2024.1266278 (PMC11021582; doi:10.3389/fmed.2024.1266278)
Supplement: Supplementary file 1 [file Table_1.docx]

**Table S1** The Baseline Characteristics of Patients from the SEER dataset before balanced and the test set

|  | SEER dataset | The test set | P-value |
| --- | --- | --- | --- |
| Age(year) |  |  | 0.933 |
| Median | 67.00 | 67.00 |  |
| Interquartile Range | 16.00 | 8.00 |  |
| Race |  |  | ＜0.001 |
| American Indian | 3（0.3%） | 0（0.0%） |  |
| Asian or Pacific Islander | 44（5.1%） | 141（100.0%） |  |
| Black | 29（3.3%） | 0（0.0%） |  |
| White | 788（90.9%） | 0（0.0%） |  |
| Unknown | 3（0.3%） | 0（0.0%） |  |
| Sex |  |  | 0.653 |
| female | 174（20.1%） | 26（18.4%） |  |
| male | 693（79.9%） | 115（81.6%） |  |
| Marriage |  |  | ＜0.001 |
| Divorced | 79（9.1%） | 0（0.0%） |  |
| Married | 572（66.0%） | 140（99.3%） |  |
| Separated | 6（0.7%） | 0（0.0%） |  |
| Never married | 92（10.6%） | 0（0.0%） |  |
| Widow | 88（10.1%） | 1（0.7%） |  |
| Unknown | 30（3.5%） | 0（0.0%） |  |
| Differentiation^1^ |  |  | 0.081 |
| 1（well） | 136（15.7%） | 18（12.8%） |  |
| 2（moderately） | 447（51.6%） | 87（61.7%） |  |
| 3（poorly） | 284（32.8%） | 36（25.5%） |  |
| Extension^2^ |  |  | 0.371 |
| Intramucosal | 373（43.0%） | 55（39.0%） |  |
| Submucosal | 494（57.0%） | 86（61.0%） |  |
| Tumor Size(mm) |  |  | 0.053 |
| Median | 17.00 | 20.00 |  |
| Interquartile Range | 17.00 | 17.50 |  |
| LNM^3^ |  |  | 0.001 |
| 0 | 711（82.0%） | 131（92.9%） |  |
| 1 | 156（18.0%） | 10（7.1%） |  |

^1^For the Differentiation variable, well-differentiated is defined as 1, moderately-differentiated is defined as 2, and poorly-differentiated or undifferentiated is defined as 3.

^2^The variable Extension refers to the depth of tumor invasion.

^3^For the LNM variable, those with lymph node metastases are defined as 1, the rest are defined as 0.
