# Supplementary material for: The development and validation of automated machine learning models for predicting lymph node metastasis in Siewert type II T1 adenocarcinoma of the esophagogastric junction
Source: Front Med (Lausanne). 2024 Apr 3;11:1266278. doi: 10.3389/fmed.2024.1266278 (PMC11021582; doi:10.3389/fmed.2024.1266278)
Supplement: Supplementary file 2 [file Table_2.docx]

**Table S2** The Clinicopathological Characteristics of Patients from the SEER dataset before balanced

|  | Total | Non-LNM | LNM | P-value |
| --- | --- | --- | --- | --- |
| Age(year) |  |  |  | 0.193 |
| Median | 67.00 | 67.00 | 65.00 |  |
| Interquartile Range | 16.00 | 16.00 | 15.00 |  |
| Race |  |  |  | 0.602 |
| American Indian | 3（0.3%） | 2（0.3%） | 1（0.6%） |  |
| Asian or Pacific Islander | 44（5.1%） | 37（5.2%） | 7（4.5%） |  |
| Black | 29（3.3%） | 23（3.2%） | 6（3.8%） |  |
| White | 788（90.9%） | 647（91.0%） | 141（90.4%） |  |
| Unknown | 3（0.3%） | 2（0.3%） | 1（0.6%） |  |
| Sex |  |  |  | 0.040 |
| female | 174（20.1%） | 152（21.4%） | 22（14.1%） |  |
| male | 693（79.9%） | 559（78.6%） | 134（85.9%） |  |
| Marriage |  |  |  | 0.622 |
| Divorced | 79（9.1%） | 62（8.7%） | 17（10.9%） |  |
| Married | 572（66.0%） | 469（66.0%） | 103（66.0%） |  |
| Separated | 6（0.7%） | 5（0.7%） | 1（0.6%） |  |
| Never married | 92（10.6%） | 80（11.3%） | 12（7.7%） |  |
| Widow | 88（10.1%） | 69（9.7%） | 19（12.2%） |  |
| Unknown | 30（3.5%） | 26（3.7%） | 4（2.6%） |  |
| Differentiation^1^ |  |  |  | ＜0.001 |
| 1(well) | 136（15.7%） | 127（17.9%） | 9（5.8%） |  |
| 2(moderately) | 447（51.6%） | 381（53.6%） | 66（42.3%） |  |
| 3(poorly) | 284（32.8%） | 203（28.6%） | 81（51.9%） |  |
| Extension^2^ |  |  |  | ＜0.001 |
| Intramucosal | 373（43.0%） | 328（46.1%） | 45（28.8%） |  |
| Submucosal | 494（57.0%） | 383（53.9%） | 111（71.2%） |  |
| Tumor Size(mm) |  |  |  | <0.001 |
| Median | 17.00 | 15.00 | 25.00 |  |
| Interquartile Range | 17.00 | 16.00 | 20.75 |  |

^1^For the Differentiation variable, well-differentiated is defined as 1, moderately-differentiated is defined as 2, and poorly-differentiated or undifferentiated is defined as 3.

^2^The variable Extension refers to the depth of tumor invasion.
