# Supplementary material for: The development and validation of automated machine learning models for predicting lymph node metastasis in Siewert type II T1 adenocarcinoma of the esophagogastric junction
Source: Front Med (Lausanne). 2024 Apr 3;11:1266278. doi: 10.3389/fmed.2024.1266278 (PMC11021582; doi:10.3389/fmed.2024.1266278)
Supplement: Supplementary file 3 [file Table_3.docx]

**Table S3** The Baseline Characteristics of Patients from the training set before and after balanced

|  | Before balanced | After balanced | P-value |
| --- | --- | --- | --- |
| Age(year) |  |  | 0.896 |
| Median | 67.00 | 66.00 |  |
| Interquartile Range | 15.00 | 13.00 |  |
| Race |  |  | 0.138 |
| American Indian | 3（0.5%） | 5（0.5%） |  |
| Asian or Pacific Islander | 30（4.8%） | 45（4.7%） |  |
| Black | 24（3.8%） | 63（6.6%） |  |
| White | 570（90.6%） | 830（87.5%） |  |
| Unknown | 2（0.3%） | 6（0.6%） |  |
| Sex |  |  | 0.041 |
| female | 128（20.3%） | 235（24.8%） |  |
| male | 501（79.7%） | 714（75.2%） |  |
| Marriage |  |  | 0.044 |
| Divorced | 53（8.4%） | 80（8.4%） |  |
| Married | 420（66.8%） | 582（61.3%） |  |
| Separated | 6（1.0%） | 8（0.8%） |  |
| Never married | 69（11.0%） | 98（10.3%） |  |
| Widow | 62（9.9%） | 147（15.5%） |  |
| Unknown | 19（3.0%） | 34（3.6%） |  |
| Differentiation^1^ |  |  | 0.081 |
| 1（well） | 104（16.5%） | 122（12.9%） |  |
| 2（moderately） | 313（49.8%） | 472（49.7%） |  |
| 3（poorly） | 212（33.7%） | 355（37.4） |  |
| Extension^2^ |  |  | 0.152 |
| Intramucosal | 274（43.6%） | 379（39.9%） |  |
| Submucosal | 355（56.4%） | 570（60.1%） |  |
| Tumor Size(mm) |  |  | ＜0.001 |
| Median | 17.00 | 20.00 |  |
| Interquartile Range | 17.50 | 19.00 |  |
| LNM^3^ |  |  | ＜0.001 |
| 0 | 511（81.2%） | 477（50.3%） |  |
| 1 | 118（18.8%） | 472（49.7%） |  |

^1^For the Differentiation variable, well-differentiated is defined as 1, moderately-differentiated is defined as 2, and poorly-differentiated or undifferentiated is defined as 3.

^2^The variable Extension refers to the depth of tumor invasion.

^3^For the LNM variable, those with lymph node metastases are defined as 1, the rest are defined as 0.
